# Supplementary material for: Multi‐dataset identification of innovative feature genes and molecular mechanisms in keratoconus
Source: J Cell Mol Med. 2024 Sep 19;28(18):e70079. doi: 10.1111/jcmm.70079 (PMC11412914; doi:10.1111/jcmm.70079)
Supplement: Supplementary file 2 — Material S2. [file JCMM-28-e70079-s002.zip › 7.GSEA analysis/5.RNF217-AS1/KEGG_FC_GAMMA_R_MEDIATED_PHAGOCYTOSIS.html]

Details for gene set KEGG\_FC\_GAMMA\_R\_MEDIATED\_PHAGOCYTOSIS[GSEA]

|  || Dataset | RNF217-AS1.group.cls#h\_versus\_l |
| Phenotype | group.cls#h\_versus\_l |
| Upregulated in class | l |
| GeneSet | KEGG\_FC\_GAMMA\_R\_MEDIATED\_PHAGOCYTOSIS |
| Enrichment Score (ES) | -0.39528713 |
| Normalized Enrichment Score (NES) | -1.5600224 |
| Nominal p-value | 0.024242423 |
| FDR q-value | 0.33142576 |
| FWER p-Value | 0.674 |
Table: GSEA Results Summary

  

Fig 1: Enrichment plot: KEGG\_FC\_GAMMA\_R\_MEDIATED\_PHAGOCYTOSIS      
 Profile of the Running ES Score & Positions of GeneSet Members on the Rank Ordered List

  

| SYMBOL | TITLE | RANK IN GENE LIST | RANK METRIC SCORE | RUNNING ES | CORE ENRICHMENT || 1 | PRKCG | na | 203 | 0.657 | 0.0478 | No |
| 2 | PIK3R5 | na | 1058 | 0.434 | 0.0586 | No |
| 3 | HCK | na | 2257 | 0.330 | 0.0509 | No |
| 4 | PLA2G4E | na | 4967 | 0.218 | -0.0095 | No |
| 5 | ARPC4 | na | 6592 | 0.165 | -0.0430 | No |
| 6 | PIK3CD | na | 6778 | 0.159 | -0.0353 | No |
| 7 | NCF1 | na | 8249 | 0.122 | -0.0678 | No |
| 8 | VASP | na | 8628 | 0.113 | -0.0695 | No |
| 9 | PIKFYVE | na | 8727 | 0.111 | -0.0632 | No |
| 10 | LIMK1 | na | 8938 | 0.106 | -0.0606 | No |
| 11 | WASF2 | na | 9032 | 0.104 | -0.0547 | No |
| 12 | VAV2 | na | 9125 | 0.103 | -0.0490 | No |
| 13 | MARCKSL1 | na | 9148 | 0.102 | -0.0413 | No |
| 14 | PIP5K1A | na | 9172 | 0.102 | -0.0336 | No |
| 15 | MYO10 | na | 9502 | 0.095 | -0.0354 | No |
| 16 | SPHK1 | na | 11035 | 0.069 | -0.0739 | No |
| 17 | DNM1L | na | 11054 | 0.069 | -0.0688 | No |
| 18 | FCGR2A | na | 11203 | 0.067 | -0.0676 | No |
| 19 | PIK3CB | na | 11439 | 0.064 | -0.0692 | No |
| 20 | DNM2 | na | 12526 | 0.048 | -0.0966 | No |
| 21 | RAC2 | na | 12945 | 0.043 | -0.1051 | No |
| 22 | ASAP3 | na | 13360 | 0.038 | -0.1140 | No |
| 23 | MAPK1 | na | 13362 | 0.038 | -0.1109 | No |
| 24 | ARPC5 | na | 13504 | 0.036 | -0.1121 | No |
| 25 | PLCG1 | na | 13642 | 0.035 | -0.1132 | No |
| 26 | GSN | na | 13780 | 0.033 | -0.1145 | No |
| 27 | PIK3CA | na | 14021 | 0.031 | -0.1189 | No |
| 28 | MAPK3 | na | 14127 | 0.029 | -0.1195 | No |
| 29 | FCGR1A | na | 14177 | 0.029 | -0.1186 | No |
| 30 | PLD2 | na | 14408 | 0.026 | -0.1232 | No |
| 31 | WAS | na | 14820 | 0.021 | -0.1333 | No |
| 32 | CFL1 | na | 15227 | 0.017 | -0.1437 | No |
| 33 | ARPC5L | na | 15307 | 0.016 | -0.1447 | No |
| 34 | ASAP2 | na | 15362 | 0.015 | -0.1450 | No |
| 35 | ARPC1A | na | 15387 | 0.015 | -0.1444 | No |
| 36 | ARPC3 | na | 15554 | 0.013 | -0.1481 | No |
| 37 | PRKCD | na | 15568 | 0.013 | -0.1474 | No |
| 38 | AKT2 | na | 15816 | 0.011 | -0.1537 | No |
| 39 | PIK3R1 | na | 16145 | 0.007 | -0.1625 | No |
| 40 | RPS6KB2 | na | 16186 | 0.007 | -0.1631 | No |
| 41 | PLA2G6 | na | 16529 | 0.003 | -0.1727 | No |
| 42 | ARPC2 | na | 16784 | 0.001 | -0.1800 | No |
| 43 | PLA2G4A | na | 17031 | -0.002 | -0.1869 | No |
| 44 | LAT | na | 17047 | -0.002 | -0.1872 | No |
| 45 | DNM3 | na | 17660 | -0.008 | -0.2042 | No |
| 46 | RPS6KB1 | na | 17704 | -0.008 | -0.2048 | No |
| 47 | WASL | na | 17958 | -0.010 | -0.2113 | No |
| 48 | CDC42 | na | 18054 | -0.011 | -0.2131 | No |
| 49 | AKT1 | na | 18280 | -0.013 | -0.2185 | No |
| 50 | RAC1 | na | 18357 | -0.014 | -0.2195 | No |
| 51 | INPP5D | na | 18494 | -0.015 | -0.2222 | No |
| 52 | PIP5K1C | na | 18811 | -0.018 | -0.2298 | No |
| 53 | RAF1 | na | 18969 | -0.020 | -0.2328 | No |
| 54 | PIP4K2B | na | 20260 | -0.033 | -0.2673 | No |
| 55 | ARF6 | na | 20937 | -0.039 | -0.2837 | No |
| 56 | CRK | na | 21000 | -0.040 | -0.2822 | No |
| 57 | FCGR2B | na | 21452 | -0.045 | -0.2916 | No |
| 58 | PLD1 | na | 21493 | -0.045 | -0.2890 | No |
| 59 | SCIN | na | 22406 | -0.055 | -0.3109 | No |
| 60 | PIK3R2 | na | 22894 | -0.061 | -0.3200 | No |
| 61 | MAP2K1 | na | 23215 | -0.064 | -0.3240 | No |
| 62 | PAK1 | na | 23429 | -0.067 | -0.3247 | No |
| 63 | PRKCA | na | 23805 | -0.072 | -0.3296 | No |
| 64 | CRKL | na | 23841 | -0.073 | -0.3246 | No |
| 65 | LIMK2 | na | 23992 | -0.075 | -0.3228 | No |
| 66 | MARCKS | na | 24237 | -0.079 | -0.3235 | No |
| 67 | SYK | na | 24484 | -0.082 | -0.3238 | No |
| 68 | VAV3 | na | 24686 | -0.086 | -0.3226 | No |
| 69 | PLCG2 | na | 24806 | -0.087 | -0.3190 | No |
| 70 | LYN | na | 24892 | -0.089 | -0.3142 | No |
| 71 | PLA2G4F | na | 24899 | -0.089 | -0.3071 | No |
| 72 | PTPRC | na | 25329 | -0.096 | -0.3117 | No |
| 73 | WASF1 | na | 25613 | -0.100 | -0.3117 | No |
| 74 | WASF3 | na | 26432 | -0.115 | -0.3259 | No |
| 75 | ARPC1B | na | 27547 | -0.138 | -0.3468 | No |
| 76 | ASAP1 | na | 27555 | -0.138 | -0.3357 | No |
| 77 | DNM1 | na | 27557 | -0.138 | -0.3245 | No |
| 78 | GAB2 | na | 28273 | -0.155 | -0.3325 | No |
| 79 | PLA2G4D | na | 28563 | -0.164 | -0.3274 | No |
| 80 | PIK3CG | na | 30335 | -0.218 | -0.3608 | No |
| 81 | PRKCE | na | 31530 | -0.265 | -0.3737 | Yes |
| 82 | FCGR2C | na | 31803 | -0.279 | -0.3587 | Yes |
| 83 | AKT3 | na | 31832 | -0.281 | -0.3365 | Yes |
| 84 | SPHK2 | na | 32339 | -0.312 | -0.3257 | Yes |
| 85 | PIK3R3 | na | 32895 | -0.352 | -0.3130 | Yes |
| 86 | FCGR3A | na | 32899 | -0.353 | -0.2843 | Yes |
| 87 | AMPH | na | 33919 | -0.466 | -0.2756 | Yes |
| 88 | VAV1 | na | 34083 | -0.499 | -0.2396 | Yes |
| 89 | DOCK2 | na | 34118 | -0.506 | -0.1993 | Yes |
| 90 | PRKCB | na | 34175 | -0.520 | -0.1584 | Yes |
| 91 | CFL2 | na | 34644 | -0.790 | -0.1075 | Yes |
| 92 | PIP5K1B | na | 34727 | -1.347 | 0.0000 | Yes |
Table: GSEA details [plain text format]

  

Fig 2: KEGG\_FC\_GAMMA\_R\_MEDIATED\_PHAGOCYTOSIS      
 Blue-Pink O' Gram in the Space of the Analyzed GeneSet

  

Fig 3: KEGG\_FC\_GAMMA\_R\_MEDIATED\_PHAGOCYTOSIS: Random ES distribution      
 Gene set null distribution of ES for **KEGG\_FC\_GAMMA\_R\_MEDIATED\_PHAGOCYTOSIS**
